# MicroRNAs and rs1803274 SNP-based BuChe downregulation are associated with metabolic syndrome through ghrelin hydrolysis and expression quantitative trait loci regulation in PD patients

**DOI:** 10.3389/fnmol.2025.1635201

**Published:** 2025-11-10

**Authors:** Guenson Chevalier, Lucas Udovin, Matilde Otero-Losada, Sofia Bordet, Santiago Perez-Lloret, Francisco Capani

**Affiliations:** 1 Centro de Altos Estudios en Ciencias Humanas y de la Salud, Universidad Abierta Interamericana, Consejo Nacional de Investigaciones Científicas y Técnicas, Buenos Aires, Argentina; 2 Centro de Investigaciones en Psicología y Psicopedagogía, Facultad de Psicología y Psicopedagogía, Pontificia Universidad Católica Argentina, Buenos Aires, Argentina; 3 Laboratorio de Investigación en Ciencia de Datos, Vicerrectorado de Investigación e Innovación Académica, Pontificia Universidad Católica Argentina, Consejo Nacional de Investigaciones Científicas y Técnicas, Buenos Aires, Argentina; 4 Facultad de Medicina, Departamento de Fisiología, Universidad de Buenos Aires, Buenos Aires, Argentina; 5 Instituto de Ciencias Biomédicas, Facultad de Ciencias de la Salud, Universidad Autónoma de Chile, Santiago, Chile

**Keywords:** single-nucleotide polymorphism, Parkinson’s disease, metabolic syndrome, microARN regulation, expression quantitative trait loci, microRNA quantitative trait loci

## Abstract

**Introduction:**

Metabolic syndrome (MetS) and Parkinson’s disease (PD) share common pathophysiological and molecular impairments related to high PD incidence in MetS patients. In this study, we searched for independently MetS-associated single-nucleotide polymorphism variants (SNVs) in PD patients and aimed to explain the molecular mechanism involved.

**Methods:**

We included 423 PD patients diagnosed by positron emission tomography (PET). A logistic regression model, the chi-squared analysis, and Fisher’s exact test were applied to additive, dominant, and recessive genetic models of data obtained from the Parkinson’s Progression Marker Initiative (PPMI) database. MicroRNA Quantitative trait Loci (MirQTL) analysis and microRNA binding to 5′/3′- untranslated regions (UTR) and conding sequence (CDS) region gene prediction analysis were performed. Expression quantitative trait loci mapping (eQTL) and gene prioritization using weighted co-expression network analysis were used to evaluate the molecular mechanisms. Chromosomal loci that explain variance in expression traits are referred to as eQTLs.

**Results:**

The SNV variant rs1803274 was associated with MetS, increased cardiovascular risk, and altered butyrylcholinesterase levels. Eleven microRNAs binding to the BuChe 3′/‘5-UTR and CDS region downregulated its expression. The rs1803274 variant was significantly enriched for neurotransmitter clearance, ghrelin secretion and deacylation, phosphatidylcholine synthesis, glycerophospholipid and lipid metabolism, and synaptic transmission. Forty-six eQTL proteins were associated with the SNV rs1803274. Thirteen of these were prioritized as potential therapeutic targets in a principal component analysis based on node degree parameters, betweenness centrality, and closeness centrality.

**Conclusion and interpretation:**

The SNV variant rs1803274 was associated with both MetS and PD and downregulated the expression of BuChe, which is involved in ghrelin hydrolysis. This variant was associated with several MetS-related eQTLs proteins or their components.

## 1 Introduction

Metabolic syndrome (MetS) is characterized by the concurrence of clinical and biochemical conditions, such as high blood pressure (BP); diabetes mellitus (DM); and/or hyperglycemia, hyperinsulinemia, and insulin resistance; central obesity; and dyslipidemia (Zhang and Tian, 2014). In 2001, the National Cholesterol Education Program (NCEP) Adult Treatment Panel III (ATP III) defined MetS as the cluster of at least three of the following: abdominal circumference over 40 in in men or 35 inches in women, BP over 130/85 mm Hg, triglyceridemia over 150 mg/dL, and fasting high-density lipoprotein cholesterol level in blood (HDL-C) under 40 mg/dL in men or 50 mg/dL in women. The International Diabetes Foundation adds insulin resistance and central obesity to satisfy a positive MetS diagnosis (Grundy et al., 2005). MetS increases cardio-cerebrovascular risk, including acute myocardial infarction and stroke, and, hence, global mortality (Malik et al., 2004; Otero-Losada et al., 2023; Etchegoyen et al., 2018).

Parkinson’s disease (PD) is a neurodegenerative disorder defined by the progressive loss of dopaminergic substantia nigra pars compacta cells, neuronal alpha-synuclein deposition, oxidative stress, and mitochondrial dysfunction. Several authors have noted associations between PD and MetS from a clinical perspective. Nam et al. studied 17,163,560 individuals and found that MetS patients were at a higher risk for PD compared to those without MetS (Malik et al., 2004; Nam et al., 2018). Age also increased PD risk further in MetS patients, as among these patients, those aged over 65 years had a higher risk of PD compared to those under 65 years (Nam et al., 2018). In a 7-year follow-up study of 85,530 participants, individuals with MetS had a 1.23-fold higher PD incidence than those without MetS (Peng et al., 2021; Roh et al., 2021). A systematic review of 11 articles involving 23,586,349 individuals found that MetS increased PD risk (Nam et al., 2018; Roh et al., 2021; Souza et al., 2021). MetS was associated with the progression of bradykinesia, rigidity, and tremor by twofold (Peng et al., 2021). MetS and PD have been reported to share mitochondrial dysfunction, oxidative stress, inflammation, and hypoxia (Jha et al., 2017). In this study, we screened single-nucleotide polymorphism variants’ (SNPVs’) loci to identify those shared by MetS and PD. Transcriptomic, microRNA binding prediction, and co-expression network analysis allowed us to determine the molecular mechanisms involved.

After identifying associations between genetic variants and MetS in PD patients, we investigated the mechanisms explaining how these variants may influence molecular process dysfunction. Genetic variants alter the expression of genetic products, such as mRNA and miRNA. Expression quantitative trait locus mapping (eQTL) identified genes specifically affected by the risk allele, enabling us to study pathways and processes associated with this variant through network analysis, hub gene enrichment, and related approaches. EQTL mapping reveals how a genetic variant affects the expression of individual genes or groups of genes. Complementary enrichment analyses of these genes highlight disrupted signaling or metabolic pathways. MiR-QTL analysis also helps determine how genetic variants influence the microRNA expression. MicroRNAs exert several functions, notably post-transcriptional mRNA degradation.

## 2 Materials and methods

### 2.1 Participants

The Parkinson’s Progression Marker Initiative (PPMI) is an ongoing multi-center observational study focused on identifying biomarkers in PD patients attending 33 clinical centers around the world (Parkinson Progression Marker Initiative, 2011). The protocol received approval from the review board at each center, and all participants provided their written informed consent. The information was shared blindly with involved and uninvolved investigators. Only data from each participant’s initial visit (baseline data) was retrieved from the PPMI database. We selected 423 participants over 30 years old diagnosed with PD based on dopamine receptor deficiency by positron emission tomography (PET) analysis.

We selected 423 subjects for the study, comprising 92 cases with MetS (21.75%) and 331 controls without MetS (78.25%), according to the MetS classification criteria established by the National Cholesterol Education Program (NCEP) Adult Treatment Panel III (ATP III) and the International Diabetes Foundation. All individuals presenting at least three of the following criteria were categorized as having MetS: abdominal circumference > 40 in in men and 35 in in women, blood pressure > 130/85 mm Hg, triglyceridemia > 150 mg/dL, fasting high-density lipoprotein cholesterolemia (HDL-c) < 40 mg/dL in men and < 50 mg/dL in women, insulin resistance, and central obesity.

### 2.2 The PPMI (Parkinson’s Progression Markers Initiative)

The Parkinson’s Progression Marker Initiative (PPMI) is a landmark observational study aimed at identifying biomarkers of PD onset and progression to facilitate the development of new diagnostic and therapeutic approaches for PD patients. It started in 2010 as an international, longitudinal study designed to establish biomarker-defined cohorts and identify clinical, imaging, genetic, and biospecimen markers of PD progression to improve disease-modifying therapeutic trials.

At PPMI’s clinical sites across 12 countries, over 2,000 participants have contributed data and biospecimens. To date, over 4,000 volunteers, including 2,000 prodromal participants from nearly 50 international sites, have entered the study. At the baseline initial visit, all participants undergo comprehensive data collection, which includes blood extractions and other biosamples for biochemical, genomic, and transcriptomic analyses, with clinical and cognitive evaluations and imaging studies. The PPMI dataset encompasses more than a decade’s worth of longitudinal and multi-modal data from patients, healthy controls, and at-risk individuals, including imaging, clinical, cognitive, and “omics” biospecimens. For all PPMI studies, each participating center receives approval from institutional ethics committees and informed consent from all participants before study participation. Our MetS-specific research focuses on baseline (first visit) data and biospecimens.

The Foundational Data Initiative for PD (FOUNDIN-PD) and Parkinson’s Progression Markers Initiative (PPMI) have a direct and complementary relationship. FOUNDIN-PD generated a multi-layered molecular dataset in a cohort of induced pluripotent stem cell (iPSC) lines from the PPMI study differentiated into dopaminergic (DA) neurons, a major affected cell type in PD. FOUNDIN-PD used 98 induced pluripotent stem cell (iPSC) lines derived from PPMI study participants, including both people with PD and unaffected individuals. PPMI provides the clinical and demographic foundation, while FOUNDIN-PD adds the cellular and molecular mechanistic layer, creating a more complete picture for researchers studying PD progression and potential therapeutic targets.

### 2.3 Genomics data analysis

Blood was drawn, and whole-genome sequencing was performed using a Macrogen Inc. sequencer on whole blood-extracted DNA samples (Parkinson Progression Marker Initiative, 2011). Each DNA sample (1 μg) was fragmented with the Covaris System and prepared following the Illumina TruSeq DNA Sample preparation guide to obtain a final library of 300–400 bp (base pair) average insert size. Alleles of the 72 variants available in the PPMI database associated with an increased risk of PD were considered. (Chevalier et al., 2023; Nalls et al., 2019) We focused on SNPs with a minimum call rate of 95%, a minor allele frequency (MAF) > 1%, and Hardy–Weinberg equilibrium *p*-values of > 0.05.

### 2.4 Transcriptomics data analysis

Ninety-eight dopaminergic cell lines derived from iPSCs of subjects enrolled in the study were obtained from the PPMI database. The FOUND-PD project used them for transcriptomic analyses, including microRNA profiling. In our study, we used normalized expression data from mRNA and microRNA transcripts already available in the PPMI database.

Whole transcriptome bulk RNA sequence was extracted from iPSCs-derived dopaminergic cells from the FOUNDIN_PD project (Bressan et al., 2023). Library preparation was performed using the SMARTer stranded total RNA sample prep kit, which has the first strand as the sense strand. Libraries—100 (base pair) bp x 100 bp—were sequenced on a NovaSeq 6,000. Fastq datasets were used to quantify coding and non-coding RNA at the gene and transcript levels. GENCODE 29 (Harrow et al., 2012) was used for transcriptomic annotation and analysis. We obtained normalized mRNA counts from the PPMI database. Out of 45,755 genes obtained—messenger ribonucleic acid (mRNA) and small non-coding RNA (SncRNA)—3,749 mRNAs were selected after data transformation and a principal component analysis (PCA)-based dimensionality reduction using the PCA node on Knime V 4.7.1.

### 2.5 MicroRNAomics data analysis

MicroRNA pools were extracted from peripheral blood. The microRNAs were sequenced in Hudson Alpha’s genomic Lab on an Illumina NovaSeq6000. Samples were prepared for library construction using a Bioo single-molecule RNA (smRNA) library prep kit. The adapters and the four random nucleotides in the 5′ ends were removed using MiRMaster (Fehlmann et al., 2021; Fehlmann et al., 2017).

The reads were mapped against the GRCh38 reference genome using Bowtie v 1.1.12. To quantify the expression of the microRNAs, reads were mapped to MirBasev22 (Griffiths-Jones, 2006; Kozomara et al., 2019; Griffiths-Jones, 2006; Kozomara et al., 2019) precursors with Bowtie and processed with MirMaster (Fehlmann et al., 2021) to allow up to one mismatch and two nucleotide overlaps at the 5′ end and five nucleotide overlap at the 3′ end of the miRNA annotation. Data were retrieved from the PPMI platform and expressed as normalized reads per million (nRPM) for the present study.

### 2.6 Statistical analysis

Quantitative data were expressed as the mean ± standard deviation, while categorical data were expressed as percentage. The chi-squared and *t*-tests were used to assess differences in clinical and biochemical variables between patients with MetS and those without MetS. To evaluate SNPs–MetS associations, we performed the chi-squared and the Fisher Exact tests using the KNIME platform V 4.7.1. A *p*-value of < 0.05 was considered significant. Hardy–Weinberg equilibrium in controls and cases was evaluated using R V4-3-0. The study included 92 cases with MetS (21.75%), 331 controls without MetS (78.25%), with *α* = 0.05, expected OR ≥ 2, and an MAF of 0.10, rendering statistical power > 85%.

### 2.7 Annotation and enrichment analysis

The SNP Nexus (Oscanoa et al., 2020) application was used to perform annotations of the SNPs–MetS associations. The GRCh37/hg19 genome was considered as the reference genome. Molecular and cellular processes-based enrichment analyses were performed using the reactome from within the SNP Nexus platform. (Oscanoa et al., 2020) This analysis allowed us to spot metabolic or signaling pathways coded by genes containing SNPs–MetS associations. We considered paths as enriched when the *p*-value was < 0.05.

### 2.8 MicroRNA binding gene prediction and MirQTL analysis

We performed a prediction analysis of microRNAs bound to MetS-associated SNP-located genes using the MirWalk platform. (Sticht et al., 2018) To evaluate microRNAs’ binding capacity to mRNA genes, we chose the following MirWalk platform parameters: folding energy, seed match, accessibility, binding probability, number of pairings, binding region length, and length of the largest consecutive pairs, allowing two mismatches. To validate the prediction, we performed a mirQTL analysis using the KNIME V4.7.1 and R v 4.3.0. MirQTL allows us to evaluate the association between MetS-associated SNPs and microRNAs using a generalized linear model and an analysis of variance (ANOVA) test. Fold2Change of microRNA expression in heterozygous and homozygous genotypes for the risk allele was computed compared with homozygous for the major allele. The Benjamini and Hochberg step-up procedure was used to control for the False Discovery Rate (FDR) and adjust *p*-values. MirQTLs with a p-value of less than 0.05 were considered significant. Significant mirQTLs predicted as binding targets in the MirWalk platform analysis were considered microRNAs associated with MetS-related SNPs.

### 2.9 Expression quantitative trait loci analysis

Trans-expression quantitative trait loci (t-eQTLs) analysis using a generalized linear model and the ANOVA test were performed to evaluate the genes whose expression was associated with MetS-related SNPs. Fold2Change for gene expression in the risk allele heterozygous and homozygous genotypes was computed compared with non-risk allele homozygous genotypes. The Benjamini and Hochberg step-up procedure was used to control for FDR and adjust *p*-values. Trans-eQTLs with a p-value of less than 0.05 were considered significant. Only eQTLs with between-genotype expression differences were selected for further analysis (ANOVA *p*-values < 0.05).

### 2.10 EQTLs enrichment, network analysis, and prioritization

Significant eQTLs differentially expressed between genotypes (ANOVA *p*-value < 0.05) were used for discovering enriched functionally related gene groups and analyzing cluster redundant annotation terms in the DAVID platform (Sherman et al., 2022; Huang et al., 2009). The co-expression matrix of significant eQTL genes was used to perform a network analysis based on weighted correlation coefficients using Cytoscape. Correlation coefficients > 0.5 and < −0.5 and with an FDR of < 0.05 were considered in the network analysis. Clustering analysis was used using several clustering algorithms offered by the Cytoscape Autoannotate Plugin, such as the Markov clustering algorithm (MCL) based on stochastic flow simulation in graphs (Van Dongen, 2008) and Affinity propagation clustering (Frey and Dueck, 2007). The eQTLs were prioritized using node degrees, closeness centrality, and betweenness centrality parameters in a principal component analysis (PCA).

## 3 Results

### 3.1 Clinical characteristics of PD patients with and without MetS

MetS was associated with hypertension (*p* < 0.00015), hypercholesterolemia (1.41 × 10–^8^), and Type II Diabetes Mellitus (DM2) (*p* < 5.45 × 10^−10^) (Table 1). In addition, BMI (*p* < 1.42 × 10^−9^), systolic BP (*p* < 0.002), and triglyceridemia (*p* < 0.00028) values were higher in PD patients with MetS than those without MetS. Total cholesterolemia and LDL values showed no between-group differences. Regardless of sex, HDL values were lower in PD patients with MetS compared to those without MetS (*p* < 0.0002) (Table 1). Age at PD onset was higher in patients with MetS than those without MetS (*p* < 0.023) (Table 1).

**Table 1:** Clinical characteristics of PD patients with and without MetS.

Variable	MetS (*N* = 92, 21.75%)	No MetS (*N* = 331, 78.25%)	*p*-value
Sex (Male/Female)	76 (82.6%)/16 (17.4%)	202 (61.02%) / 129 (38.98%)	0.0001
Hypertension (Yes/No)	30 (32.6%)/62 (67.4%)	50 (15.11%)/281 (84.89%)	0.0001
Systolic BP (mm Hg) (mean ±SD)	135.77 ± 15.65	129.56 ± 17.21	0.0020
Diastolic BP (mm Hg) (mean ±SD)	79.17 ± 10.60	77.22 ± 10.89	0.1200
Hypercholesterolemia (Yes/No)	24 (26.1%)/68 (73.90%)	17 (5.14%)/314 (94.86%)	1.41E^−8^
Type II diabetes mellitus (Yes/No)	13 (14.13%)/79 (85.87%)	2 (0.6%)/329 (99.4%)	5.45E^−10^
Total HDL (Mean ± SD)	46.81 ± 14.91	62.80 ± 18.66	1.02E^−7^
HDL (Male) (mean ± SD)	45.95 ± 15.53	56.91 ± 14.06	0.0002
HDL (Female) (mean ± SD)	51 + −10.95	71.50 + −21.25	0.0002
Triglyceridemia (mean ± SD)	133.88 ± 68.28	102.02 ± 37.89	0.0003
LDL (mean ±SD)	106.49 ± 41.73	107.73 ± 32.39	0.8500
Total cholesterolemia	180.46 ± 48.48	191.83 ± 39.38	0.1100
BMI (kg/m^2^)	29.72 ± 4.32	26.41 ± 4.47	1.42E^−9^
Age at PD onset (years) (mean ± SD)	63.06 ± 8.82	60.62 ± 9.86	0.0230

Between-group differences (with vs without MetS) were analyzed using the Chi-square test (categorical variables) or the Student’s *t*-test (numerical variables). *p* < 0.05: significant between-group difference.

### 3.2 SNP rs1803274-MetS association revealed by genetic additive and dominant model

Only the SNP rs1803274 located in the BuCHe gene was associated with MetS (Table 2).

**Table 2:** SNPs associated with MetS by *χ*^2^ (Chi square) test analysis.

SNP Locus name	*χ*^2^	*χ*^2^ *p*-value
BuChE_rs1803274	7.94	0.018
NOTCH4_G1739S_rs8192591	4.98	0.082
LRRK2_R1628P/H_rs33949390	2.69	0.100
COMT_rs174674	4.41	0.109
DDRGK1_rs55785911	3.74	0.154
BST1_rs11724635	3.46	0.176
NUCKS1_rs823118	4.85	0.182
SREBF1_rs11868035	3.37	0.184
ZNF184_rs9468199	4.58	0.205
COMT_rs165656	3.07	0.214
ANK2/CAMK2D_rs78738012	4.26	0.233
SNCA_rs356181	2.89	0.234
MIR4682_rs118117788	1.37	0.240
LRRK2_rs76904798	2.69	0.260
COMT_rs4633	2.49	0.287
MAPT_rs17649553	3.55	0.313
PMVK_rs114138760	0.96	0.326
TMEM175_rs34311866	2.15	0.340

Additive model.

*χ*^2^ test was performed to identify SNPs-MetS associations. Nominal *p* values were analyzed. Nomenclature shows the gene where the SNP is located, followed by an underscore (_) and the SNP name itself—numbers preceded by “rs.” Some names include information about the protein mutations—position and amino acid change.

We found a significant association between MetS and SNP rs1803274 ^1^ using the dominant genetic model (Table 3). The SNP rs1803274 BuChe was associated with MetS (*p* = 0.018, Chi-Square Test, additive genetic model) (Supplementary Figure 1, Table 2). SNP rs1803274-MetS association was significant using the dominant model (Table 3). Hence, the single appearance of the risk allele (minor allele) SNP rs1803274 was associated with MetS. Using the SNP nexus application, we identified significant enrichment of SNP rs1803274 for processes involved in neurotransmitter clearance, ghrelin synthesis, secretion and deacetylation, phosphatidylcholine synthesis, glycerophospholipid and lipid metabolism, and synaptic transmission (Table 4).

**Table 3:** SNPs-MetS association as by dominant and recessive model and *χ*^2^ (Chi-square) test analysis.

Model	*χ*^2^	*χ*^2^ *p*-value	*F*-Test *p*-value
Dominant	6.71	0.009	0.009
Recessive	3.36	0.06	0.08

This table presents the association analyses using the dominant and recessive model for the SNP rs1803274 associated with MetS according to the additive model. Nominal *p*-values were analyzed.

**Table 4:** Molecular and cellular process enrichment for MetS-associated SNP rs1803274.

Molecular process	*P*-value	Adjusted *P*-value
Neurotransmitter clearance	0.0009	0.0102
Ghrelin synthesis, secretion, and deacetylation	0.0017	0.0097
PC (phosphatidylcholine) synthesis	0.0026	0.0095
Peptide hormone metabolism	0.0083	0.0230
Glycerophospholipid biosynthesis	0.0119	0.0262
Phospholipid metabolism	0.0196	0.0359
Transmission across chemical synapses	0.0249	0.0391
Neuronal system	0.0381	0.0524
Lipid metabolism	0.0685	0.0837
Protein metabolism	0.0188	0.2070
Metabolism	0.0194	0.1940

This table presents the results of the enrichment analysis of the MetS-associated SNP rs1803274 located at the BuChe gene. Adjusted *p*-values were calculated using the Benjamini and Hochberg technique for Type I error correction in multiple test analysis.

### 3.3 Annotation and protein prediction effect of the MetS-associated SNPs rs1803274

SNP rs1803274 located on chromosome 3q26.1 had an overall MAF of 0.16 using the SNPnexus online platform. (Oscanoa et al., 2020) It is a non-synonymous SNP located in the CDS region at position 1,699 of the BuChe gene (results found in SNP nexus on 13/3/2023). There was a change of the non-polar Ala (A, alanine) to the polar Thr (T, threonine) in position 567 according to Ensembl, RefSeq, UCSC, and CCDS data (Oscanoa et al., 2020; Cunningham et al., 2022; Howe et al., 2021; O’Leary et al., 2016; Nassar et al., 2023; Lee et al., 2022; Pujar et al., 2018; Farrell et al., 2014). The pathogenicity of rs1803274 SNP was predicted by the SIFT (Ng and Henikoff, 2001; Ng and Shift, 2003; Sim et al., 2012) and Polyphen (Adzhubei et al., 2010) applications as benign and tolerable. In both the control group and the cases, the SNP rs1803274 was in Hardy–Weinberg equilibrium (*χ*^2^ *p* values 0.62 and 0.17, respectively).

### 3.4 MirQTL analysis on the association between microRNAs and the BuChe gene expression in iPSCs-derived dopaminergic cells

We performed a generalized linear model-based MirQTL analysis to study the association between normalized BuChe gene counts obtained from dopaminergic cell-derived iPSCs and normalized microRNA counts from blood cells. Data of normalized BuChe gene counts from iPSC-derived dopaminergic cells and normalized counts of microRNAs from patients’ peripheral blood cells were obtained from the PPMI database. In a workflow on the KNIME platform, we ran iterative loops on the data. In each loop, microRNA data were entered, and the workflow proceeded, merging them into a table with the BuChe gene data to be normalized using the same scale. After connecting to an R server within the KNIME platform, the generalized linear model was used. Every microRNA underwent the same procedure. Out of 43 microRNAs identified as significantly associated with the BuChe gene, 11 microRNAs showed a negative effect size (downregulation) on BuChe gene expression (Supplementary Table 5).

### 3.5 Prediction of microRNAs binding to BuChe-mRNA

To address whether some microRNAs might bind the 3′/5’-UTR and CDS region of the BuChe mRNA transcript for destruction and/or translation inhibition—thus contributing to BuChe expression deficit in patients bearing the SNP rs1803274-BuChe risk allele—we performed an in-silico binding prediction analysis between the microRNAs and the BuChe gene. We used the online miRWalk platform (www.mirwalk.umm.uni.heidelberg.de) that searches for microRNA binding within the entire mRNA sequence (5’-UTR, 3’-UTR, CDS) using data from TarPmiR, a microRNA target site prediction tool. (Ding et al., 2016) Out of 1,617 microRNAs interacting with the BuChe gene, 28 microRNAs matched those identified using the generalized linear model. Twenty-four of them were bound to the CDS region of the gene, 3 to the 5’-UTR, and 1 to the 3’-UTR region (Supplementary Table 6).

### 3.6 EQTL analysis identified 70 genes whose expression was influenced by MetS-related SNP rs1803274 genotypes

**Figure 1:**
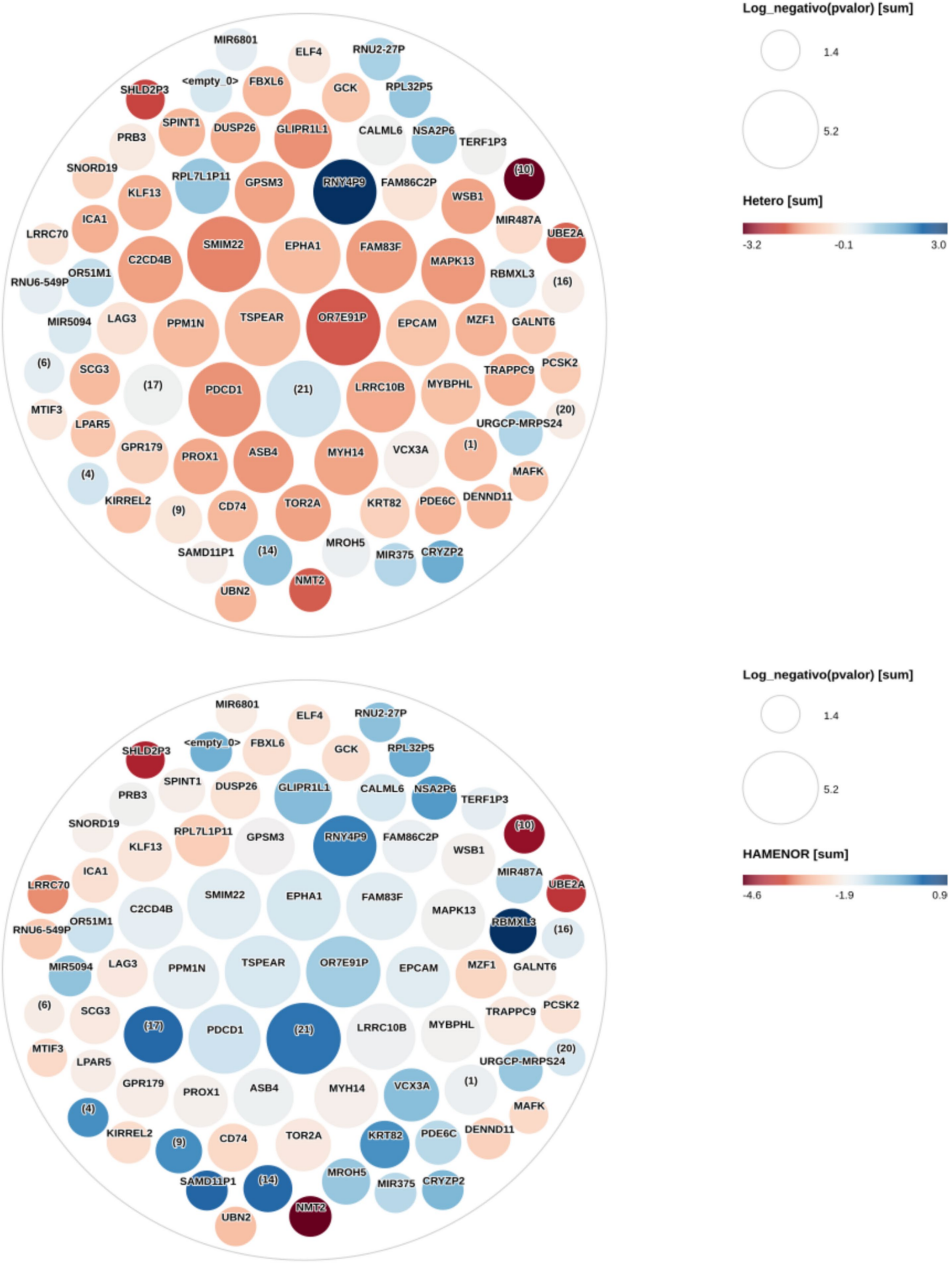
Heterozygous and homozygous risk allele effect on the eQTLs expression changes. This figure shows eQTLs expression changes (Log2 fold change) in heterozygous and homozygous forms for the risk allele compared with the non-risk homozygous allele. The circles represent the eQTLs. Circle size is proportional to the negative Log10 *p*-values. The larger the circle, the lower its *p*-value (<0.05), and the higher the signal. All eQTLs presented in this figure have a *p* value < 0.05 and an FDR ≤ 0.061. The colors represent the Log2 fold change in gene expression in the heterozygous and homozygous risk allele compared with the non-risk homozygous allele. The Log2 fold change ranges from red to blue with shades in between. Dark red represents decreased eQTL expression in the heterozygous risk allele, and dark blue represents increased eQTL expression in the homozygous risk allele. The HGNC gene symbol is shown in each circle (eQTL) center. Some circles identified by numbers in parentheses have not been mapped to any genes during the annotation analysis and are new genes yet not studied. Genes in the center of the heterozygous figure with higher signal—larger circles—are in intense red, while they are bluish or dark blue in the homozygous figure. eQTLs OR7E91P, TSPEAR, FAM83F, EPHA1, GPSM3, SMIM22, C2CD4B, PPM1N, GLIPR1L1, FAM82C2P, WSB1, MIR487A, MAPK13, EPCAM, PDCD1, LRRC10B, MYBPHL, PROX1, ASB4, PDE6C, KRT82, and SAMD11P1 have lower Log2 fold change in heterozygous (lower expression) than homozygous risk allele. MIR6801, RPL7L1P11, and RNUG-549P have lower expression in heterozygotes. No major differences were noted in RNU2-27P, RPL32P5, NSA2P6, TERF1P3, UBE2A, OR51M1, URGCP-MRPS24, CRYZP2, NIR375, MROH5 genes expression between heterozygous and homozygous risk alleles.

Dimensionality reduction analysis resulted in 3,749 mRNAs. In the generalized linear model, including SNPs genotype and normalized count BuChe gene data, we found 116 eQTLs associated with the SNPs rs1803274 (*p* < 0.05), but none were found after *α* correction due to multiple test effects. The BuChe gene was not associated with the SNP variant rs1803274 in the regression model (*β* = −0.12, *p* = 0.49). No difference in the BuChe gene expression was found between SNP rs1803274 genotypes (ANOVA, *p* = 0.76). The 116 eQTLs were used in an ANOVA analysis to study their expression according to the genotypes of the rs1803274-buChe SNP variant. We identified 70 eQTLs with significantly altered expression according to the risk heterozygous and homozygous genotype alleles compared with the non-risk homozygous genotype alleles (See Figure 1).

### 3.7 Enrichment analysis by the DAVID platform revealed 27 eQTLs known associated with MetS

**Figure 2:**
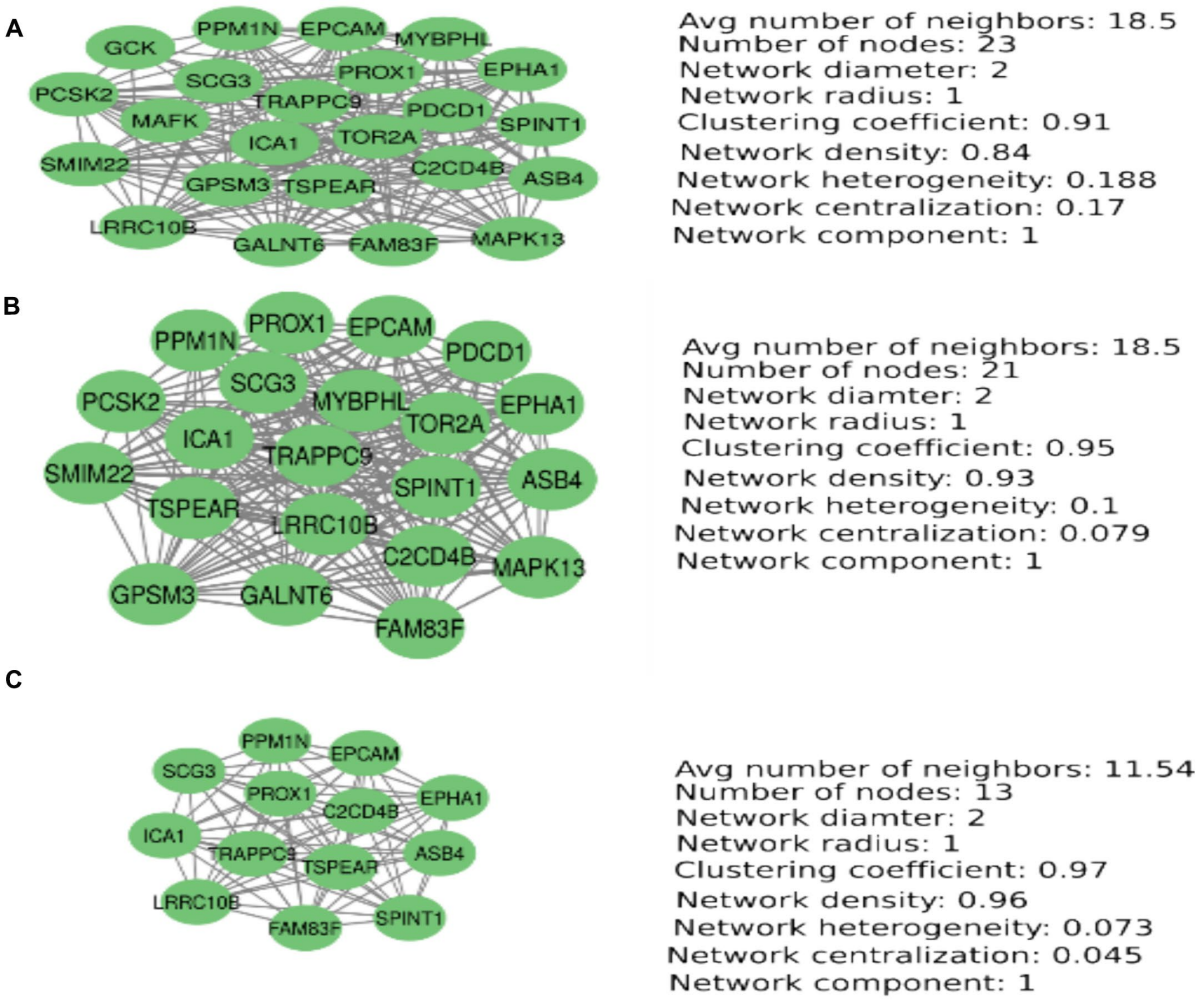
PCA-based MetS-associated eQTLs prioritization. Node degrees, betweenness, and closeness centrality to prioritize the most important eQTLs associated with MetS-related SNP rs1803274. This figure shows the prioritization of the most important nodes according to the parameters nodesdegrees, betweenness centrality, and closeness centrality. A principal component analysis was computed using these parameters in each case. **(A)** Nodes degree-based PCA. **(B)** Closeness centrality-based PCA. **(C)** Betweenness centrality-based PCA.

The 70 identified eQTLs (see above) were entered into the DAVID database for functional annotation and enrichment analysis, out of which 68 were mapped. There were 11 genes with missing information for annotation in the Ensembl platform. A 47-eQTLs cluster with an enrichment score of 15.71 (*p* = 3.5 × 10^−24^, FDR = 1.3 × 10^−20^) was computed. Twenty-one (44.68%) eQTLs from the cluster have already been identified in other studies (Supplementary Table 7) as associated with MetS or its components—DM, hypertension, obesity, and insulin resistance—(Supplementary Table 7). The other 26 eQTLs from the cluster—DENND11, FBXL6, GLIPR1L1, EPCAM, FAM83F, FAM86C2P, LRRC70, LPAR5, MROH5, MYBPHL, OR51M1, OR7E91P, GALNT6, PPM1N, SPINT1, SMIM22, TSPEAR, UBN2, UBE2A, VCX3A, PRB3, ELF4, MAFK, TORA2, CALML6, and MZF1—would be novel eQTLs associated with MetS in PD patients. Figure 2 shows how eQTL expression changes in both heterozygous and homozygous risk alleles taking as reference the non-risk homozygous allele.

### 3.8 Weighted correlation-based eQTLs related to MetS network analysis

The 116 eQTLs were used to perform a weighted-correlation network analysis with 46 significant nodes (FDR < 0.05, correlation coefficient > 0.5, and <−0.5). The network had 46 nodes, 308 interactions (edges), network diameter 5, clustering coefficient = 0.66, network density = 0.33, network heterogeneity = 0.65, and two connected components. We found four eQTLs (genes) with the highest importance: SCG3 (Hub scores = 1, authority score = 1, node degree = 29), ICA1 (Hub score = 0.98, authority score = 0.98, node degree = 27), EPHA1 (Hub score = 0.97, authority = 0.97, node degree = 27), and MAPK13 (Hub Score = 0.96, authority score = 0.96, node degree = 26). We obtained three clusters using the community cluster algorithm (CCA) of the Cytoscape Autoannotate plugin (Supplementary Figure 2). Signaling pathways and molecular processes for which these clusters were enriched are shown in Supplementary Tables 8–10. Supplementary Tables 11–13 show eQTLs expression according to SNP rs1803274 genotype.

### 3.9 Prioritization of the most important eQTLs

We have used node degree, closeness centrality, and betweenness centrality metrics to find the most important nodes in the network. The set of most important nodes was obtained from a principal component analysis for each metric (Figure 1). The principal component containing the most important nodes for the case in which the betweenness centrality metric was used contains 13 nodes. These nodes are also found when using the nodes’ degree and closeness centrality parameters. These 13 nodes are important because of their capacity to establish fast communications with other nodes in the network and the high number of connections they establish with other nodes. These nodes are strongly interconnected with high network coefficients ranging from 0.91 to 0.97(Figure 1).

## 4 Discussion

### 4.1 Several MicroRNAs bind to the BuChe gene and downregulate its expression

We found an association between SNP rs1803274-BuChe—located in the CDS region of the BuChe gene at position 1,699—and MetS in PD patients, evidenced by both additive and dominant genetic models. SNP rs1803274 risk allele was associated with decreased BuChe gene expression. MetS and obesity have been found to be associated with decreased BuChe gene expression in other studies (Li et al., 2008). EQTL analysis did not show a significant decrease in BuChE gene expression. Yet, prior studies suggested this variant reduced BuChE protein levels. MiRNA-mediated regulation may explain this discrepancy. Certain microRNAs may target and degrade BuChE transcripts, lowering protein expression. To test this hypothesis, we performed a miR-QTL analysis to identify microRNAs associated with the variant, followed by binding prediction analyses to assess whether these microRNAs could interact with the BuChE gene.

Our findings suggest that BuChe gene downregulation might result from some microRNAs binding to BuChe transcript mRNA to inhibit its translation or promote its degradation. This idea is supported by the present identification of 11 microRNAs—related to BuChe gene downregulation—that bind to the 3′/5’-UTR and CDS BuChe gene based on *an in silico* prediction analysis.

### 4.2 Downregulated BuChe is associated with upregulated acyl-ghrelin and MetS in PD patients

We identified the SNP rs1803274 variant enriches for chemical synaptic transmission, neurotransmitter clearance processes, phosphatidylcholine synthesis, glycerophospholipids, and phospholipid synthesis. BuChe expression alteration is linked to impairment in these molecular processes. The SNP rs1803274 variant also enriches for ghrelin synthesis, secretion, and deacylation. As ghrelin is a substrate for BuChe activity, rendering unacylated ghrelin, BuChe gene downregulation would lead to an increase in the acyl-to-unacylated ghrelin ratio. Ghrelin hormone is involved in hunger and satiety regulation (Nakazato et al., 2001). In rats, acyl-ghrelin IV injection increased hunger and body weight, while anti-ghrelin G antibodies produced the opposite effects (Nakazato et al., 2001). Acylated and unacylated ghrelin are associated with increased or decreased neurogenesis in the hippocampus, respectively (Hornsby et al., 2020). Acyl-ghrelin upregulated the neurogenic brain-derived neurotrophic factor (BDNF) in a promoter, age-dependent manner (Bayliss et al., 2016; Sassi et al., 2022).

### 4.3 Rs1803274-BuChe-based EQTLs and weighted co-expression network-based clustering analysis revealed gene clusters associated with metabolic syndrome and its components

We performed an eQTL analysis of the genes of iPSC-derived dopaminergic cells from PD patients. Out of 116 genes associated with SNP rs1803274, 70 eQTL were expressed depending on genotype. Only PD patients with heterozygous and homozygous genotypes for SNP rs1803274 minor allele were found at MetS risk. The DAVID enrichment analysis application computed a cluster of 47 genes—70 eQTLs entered—44.68% of which were already recognized as associated with MetS components.

A cluster analysis of a weighted co-expression network resulted in three clusters: the first enriched for processes associated with growth cone collapse and neurite outgrowth, and the second enriched for antigen degradation and presentation to T lymphocytes. CD74 presence in our data might cause cross-presentation of antigens by MHC I and participate in autoimmune DM in these patients, given the presence of autoantigen ICA1—linked to autoimmune DM—in our data.

SCG3 protein is involved in granular regulation and biogenesis in neurons and pancreatic cells. The alteration in the SCG3 gene activity or expression might affect proinsulin sorting and proteolytic processing of prohormones in pancreatic beta cells, decreasing insulinemia, and increasing DM risk. The intracellular increase in protein content might trigger cell survival or apoptotic mechanisms involving the p38/MAPK pathway, signaling to MST1, RAS, ERKs, or NOD 1/2 pathways, as found in the enrichment analysis of the third cluster.

The eQTL ubiquitin-conjugating enzyme 2A (UBE2A) was downregulated by SNP rs1803274-BuChe in the previous analysis of this study (Supplementary Table 13). This protein accepts polyubiquitin from the E1 complex at position lysine 48 and catalyzes its covalent attachment to other proteins for subsequent degradation (David et al., 2010) by the 26S proteasome complex.

There are other forms of mono-ubiquitination of different lysine residues of the target protein involving UBE2A and the 26S proteasome complex for the cellular processes of endocytosis and sorting of proteins to different cellular compartments (Hicke and Dunn, 2003).

Downregulation of UBE2A expression could affect protein degradation processes and subsequent cellular processes, such as endocytosis and sorting proteins. Downregulation of UBE2A has been associated with brain amyloid plaque accumulation in Alzheimer’s disease patients (Zhao et al., 2016). Several proteins of the proteasome system, including PARK2, ubiquitin-conjugating enzyme HIP2, and UBE2L3, are associated with PD (Su et al., 2018; Shimura et al., 2000; Zhang et al., 2022).

A mutation in the UBE2A gene was found to impair neuronal function and alter parkin gene-dependent mitophagy (Zhang et al., 2022; Haddad et al., 2013). In addition, the ubiquitin-conjugating enzyme UB2O, related to UBE2A, promotes insulin resistance and obesity in MetS animal models (Vila et al., 2019). We suggest that an SNP rs1803274-BuChe-dependent decrease in UBE2A gene expression might increase the risk of both MetS and PD.

Presently, eQTL VPS26A was downregulated by SNP rs1803274 in a regression model. VPS26A is a component of the retromer cargo-selective complex (CSC) (Priya et al., 2015).

The CSC is believed to prevent the mis-sorting of selective transmembrane cargo proteins into the lysosomal degradation pathway. Three mutations in the VPS26A protein (p. K93E, p. M112V, and p. K297X) were associated with atypical Parkinsonism (Vila et al., 2019; McMillan et al., 2016) by affecting the interaction between VPS26A and the SNX27 cargo adaptor (Gustavsson et al., 2015). Variants in the VPS26A(rs1802295) gene are associated with DM2 (Shabana et al., 2016).

In this study, DENND10—another eQTL—was downregulated by the SNP rs1803274. It is a guanine nucleotide exchange factor (GEF) that regulates the homeostasis of the endocytic pathway, including endosomal positioning, maturation, and secretion, likely activating Rab proteins like RAB27A and RAB27B. It promotes guanosine diphosphate (GDP) to guanosine triphosphate (GTP) change, converting inactive GDP-bound RAB27A and RAB27B into their active GTP-bound form. (Priya et al., 2015; Zhang et al., 2019)

## 5 Conclusion

This study found BuChe-located SNP rs1803274’s association with MetS. The BuChe gene was reported to be downregulated by the SNP rs1803274 by other authors. We have identified 28 microRNAs that bind to the BuChe mRNA in both 3′, 5’-UTR and CDS gene regions. Of these, 11 downregulated the BuChe gene expression. BuChe gene downregulation is associated with increased acyl-ghrelin expression. Ghrelin increases hunger and MetS risk and promotes neurogenesis depending on the BDNF level. We identified 116 eQTLs associated with SNP rs1803274, of which 70 have expressions influenced by SNP genotypes. The DAVID application computed a cluster of 47 eQTLs, 21 of which were already known as associated with MetS or its components. The major eQTLs prioritized in our analysis were ICA1, SCG3, EPHA1, PPM1N, EPCAM, ASB4, SPINT1, FAM83F, LRRC10B, PROX1, TRAPPC9, TSPEAR, and C2CD4B. These eQTLs were found as the best node degrees, the best betweenness centrality, and the best closeness centrality, forming a network cluster in a PCA analysis. Of them, SCG3, ICA1, and EPHA1 were the Hub eQTLs selected.

## Data Availability

Publicly available datasets were analyzed in this study. This data can be found at: https://www.ppmi-info.org/access-data-specimens/download-data.

## Footnotes

^1^SNP rs1803274 is associated with the butyrylcholinesterase (BuChE, Human Gene Nomenclature Committee, HGNC, symbol BCHE; EC 3.1.1.8) gene. The rs1803274(A) allele (CHE*539 T or BCHE*539 T) encodes the K variant of BuChE, reduces serum BuChE activity by 30–60%, and is often co-inherited with the SNP encoding the so-called “atypical” butyrylcholinesterase.
