# Supplementary material for: MicroRNAs and rs1803274 SNP-based BuChe downregulation are associated with metabolic syndrome through ghrelin hydrolysis and expression quantitative trait loci regulation in PD patients
Source: Front Mol Neurosci. 2025 Nov 10;18:1635201. doi: 10.3389/fnmol.2025.1635201 (PMC12641004; doi:10.3389/fnmol.2025.1635201)
Supplement: Supplementary file 1 [file Table_1.pdf]

Table 5. Association analysis between microRNAs and BuChe gene expression in iPSCs derived dopaminergic cells

MicroRNA name	Effect size	P value	Adjusted P value
hsa-miR-514a-3p	0.15	0.00101	0.043
hsa-miR-4445-3p	0.19	0.00108	0.023
hsa-miR-6751-3p	0.15	0.00115	0.016
hsa-miR-4719	0.17	0.00344	0.037
hsa-miR-4477a	0.17	0.00375	0.032
hsa-miR-374c-3p	0.17	0.00456	0.033
hsa-miR-4634	0.16	0.00522	0.032
hsa-miR-4521	**-0.15**	0.0103	0.055
hsa-miR-4762-3p	0.15	0.0126	0.060
hsa-miR-20b-3p	0.14	0.0142	0.061
hsa-miR-3659	0.14	0.01.57	0.061
hsa-miR-4638-5p	0.14	0.01.61	0.058
hsa-miR-3944-3p	**- 0.14**	0.01.64	0.054
hsa-miR-657	0.14	0.0176	0.054
hsa-miR-365b-5p	-0.14	0.0177	0.051
hsa-miR-6727-3p	0.14	0.0186	0.050
hsa-miR-3144-5p	0.13	0.0208	0.053
hsa-miR-6780a-5p	**-0.13**	0.02.33	0.056
hsa-miR-6130	0.13	0.0234	0.053
hsa-miR-1181	**-0.13**	0.0257	0.055
hsa-miR-6850-5p	0.13	0.0263	0.054
hsa-miR-212-5p	0.12	0.0302	0.059
hsa-miR-92a-2-5p	**-0.12**	0.0307	0.057
hsa-miR-4520-5p	0.13	0.0309	0.055
hsa-miR-3714	0.12	0.0324	0.056
hsa-miR-6801-3p	**-0.12**	0.0329	0.054
hsa-miR-6814-3p	0.12	0.0333	0.053
hsa-miR-4775	0.13	0.0334	0.051
hsa-miR-887-3p	-0.12	0.0366	0.054
hsa-miR-3139	0.12	0.0369	0.053

hsa-miR-6755-5p	0.12	0.0380	0.053
hsa-miR-624-5p	0.12	0.0390	0.052
hsa-miR-7152-5p	0.12	0.0399	0.052
hsa-miR-33b-5p	0.12	0.0401	0.051
hsa-miR-4668-5p	**-0.12**	0.04.01	0.049
hsa-miR-4433b-5p	**-0.12**	0.0402	0.048
hsa-miR-7107-3p	0.12	0.0413	0.048
hsa-miR-4676-5p	0.12	0.0418	0.047
hsa-miR-3192-5p	**-0.12**	0.0428	0.047
hsa-miR-3679-5p	**-0.11**	0.0454	0.049
hsa-miR-129-2-3p	0.11	0.0477	0.050
hsa-miR-299-3p	0.11	0.0483	0.049
hsa-miR-9902	0.11	0.0486	0.049

Table 5- Association analysis between microRNAs and the BuChe gene expression iPSCs-derived dopaminergic cells. MirQTLs. In this analysis we studied the effect of microRNAs on the BuChe gene expression where is located the MetS-associated SNP rs1803274. A generalized linear regression model was used. Data of normalized BuChe gene counts from iPSC-derived dopaminergic cells and normalized counts of microRNAs from patients' peripheral blood cells were obtained from the PPMI database. This table shows the MicroRNAs associated with the BuChe gene, the effect size, their p-Values. The Benjamini and Hochberg technique for Type I error correction were used for computing the adjusted p-Values. It was found that 11 MicroRNAs downregulates the BuChe gene expression (Negative effect size).

Table 6. Prediction of microRNAs binding to BuChe-mRNA using the miRWalk web application.

MicroRNA name	Start	End	Binding p	Energy	Acces sibility	Pairin gs	Binding region length	Longest consecutive pairings	Position
hsa-miR-92a-2-5p	487	509	0.923	-17.1	0.017	19	22	14	CDS
hsa-miR-212-5p	1281	1302	0.923	-18.7	0.001	19	21	12	CDS
hsa-miR-299-3p	893	915	0.846	-16.8	0.190	17	22	7	CDS
hsa-miR-365b-5p	28	51	1.0	-27.7	0.002	18	23	8	5’-UTR
hsa-miR-20b-3p	287	308	0.923	-18.8	0.038	17	21	11	CDS
hsa-miR-33b-5p	269	306	1.0	-20.0	0.036	17	37	8	CDS
hsa-miR-657	640	673	0.923	-21.8	0.005	15	33	8	CDS
hsa-miR-887-3p	602	654	0.846	-21.6	$2.853E^{-6}$	10	14	7	CDS

hsa-miR-1181	1362	1388	0.923	-21.7	0.001	17	26	7	CDS
hsa-miR-3139	1024	1058	0.923	-19.1	$9.518E^{-5}$	17	29	8	CDS
hsa-miR-3144-5p	153	170	0.923	-16.2	0.121	15	17	8	CDS
hsa-miR-3192-5p	270	293	1.0	-19.2	0.004	18	23	10	CDS
hsa-miR-3679-5p	71	96	0.846	-19.4	0.009	19	25	13	5'-UTR
hsa-miR-3944-3p	20	55	0.846	-30.9	0.002	20	35	12	5'-UTR
hsa-miR-4445-3p	149	178	0.923	-16.7	0.015	14	17	12	CDS
hsa-miR-4520-5p	1789	1840	0.846	-19.5	0.096	17	44	12	CDS
hsa-miR-4521	644	667	0.846	-20.8	$2.075E^{-5}$	19	23	12	CDS
hsa-miR-4634	1364	1409	0.846	-18.8	0.004	16	45	8	CDS
hsa-miR-4638-5p	839	863	0.846	-21.3	0.003	13	17	9	CDS
hsa-miR-4762-3p	373	406	1.0	-19.9	0.005	20	33	8	CDS
hsa-miR-6751-3p	673	693	0.846	-18.2	$1.277E^{-5}$	12	20	7	CDS
hsa-miR-6755-5p	642	656	0.846	-16.6	$2.798E^{-6}$	11	14	7	CDS
hsa-miR-6780a-5p	2179	2229	0.846	-19.5	0.116	20	34	15	3'-UTR
hsa-miR-6801-3p	1362	1386	0.923	-22.7	$3.088E^{-4}$	17	24	5	CDS
hsa-miR-6814-3p	1483	1510	0.923	-22.0	$5.598E^{-4}$	16	20	7	CDS
hsa-miR-6850-5p	1399	1422	0.846	-20.6	$2.001E^{-4}$	17	23	9	CDS
hsa-miR-7107-3p	1490	1522	0.923	-29.8	0.007	27	32	8	CDS

Twenty-eight microRNAs were identified in the miRwalk platform. They were associated with the BuChe gene in the linear regression analysis performed using normalized counts of the BuChe gene and microRNAs from iPSCs-derived dopaminergic and blood cells respectively. **Start:** base pairs where microRNA-mRNA binding starts. **End:** base pairs where microRNA-mRNA binding ends. **Binding p:** binding probability. Energy: minimal folding energy. **Accessibility:** The accessibility is a measurement of whether the target site region in the mRNA sequence is open for miRNA to binding. **Pairings:** Total number of paired positions. This feature is calculated as the total number of paired positions for each miRNA-mRNA binding site. **Binding region length:** This feature is calculated as the length of miRNA binding target site region. For example, if miRNA x binds to mRNA y and the binding site between x and y are 28 nts region on mRNA y, this feature is 28. **Longest consecutive pairings:** the length of the largest consecutive pairs. Of the 28 microRNAs identified, 24 are bound to the CDS region of the gene, 3 to the 5'-UTR and one to the 3'-UTR region.

Table 7. eQTLs associated with SNP rs1803274 related to MetS or its components.

Gene name	MetS or its components	References
CD74	DM2, DM1	Chen et al 2022(59), Korf et al 2017(60), Liu et al. 2022(61)
EPHA1	DM2	Li et al. 20017(62)
GPSM3	Obesity	Martin et al. 2019(63)
KLF13	Obesity	Koh et al. 2017(64)
NMT2	Obesity, DM2	Dong et al. 2021(65)
WSB1	MetS	Alsabbagh et al 2023(66)
ASB4	Obesity	Kriebs e al. 2022(67)
GCK	Insulin resistance, DM2	Li et al 2023(68), Fendler et al. 2012(69)
ICA1	Obesity, DM2	Delpero et al. 2022(70), Barkalifa et al. 2010(71)
KIRREL2	Microalbuminuria	Voskarides et al 2017(72)
LAG3	DM2	Zhang et al. 2017(73)
MTIF3	Obesity	Huang et al. 2023(74)
MAPK13	Insulin resistance, DM2	Cuenda et al. 2009(75)
PDE6C	Diabetic retinopathy	Butt et al. 2012(76)
PDCD1	DM1	Gu et al 2018(77)<br>Nyambuya et al 2021(78)
PCSK2	DM2	Chang et al. 2015(79)
PROX1	Obesity, DM2	Kretowski et al. 2015(80)
SCG3	MetS	Lin et al. 2019(81)
TRAPPC9	Hypertriglyceridemia	Ronn et al 2023(82)
DUSP26	Obesity	Jacques et al.2021(83)
MYH14	MetS	Louis et al 2022(84)

Table 7- This study presents the eQTLs associated with metabolic syndrome or its components based on results from other studies. Out of the cluster of 47 eQTLs, 21 were found to be associated with metabolic syndrome and/or its components in previous studies. DM2: type II diabetes mellitus; DM1: type I diabetes mellitus. MetS: Metabolic syndrome

Table 8- Enrichment of EQTLs Cluster-1 associated with MetS-related SNP rs1803274 using Enrichr web application

Pathway	p-value	adjusted p-value	Odds Ratio	Genes
EPH-ephrin signaling	0.0036	0.071	24.832	MYH14; EPHA1
Signaling by MST1	0.0049	0.071	262.842	SPINT1
MET receptor activation	0.0059	0.071	210.263	SPINT1
Cell surface interactions in vascular wall	0.0078	0.071	16.707	CD74; EPCAM
DSCAM interactions	0.011	0.071	105.105	MAPK13
P38 MAPK events	0.012	0.071	95.545	MAPK13
POU5F1 (OCT4), SOX2, NANOG	0.012	0.071	95.545	EPHA1
Lysosphingolipid and LPA receptors	0.014	0.071	80.838	LPAR5
Axon guidance	0.014	0.071	6.656	MYH14; EPHA1; MAPK13
EPHA-mediated growth cone collapse	0.015	0.071	75.060	MYH14
Nervous System Development	0.016	0.071	6.328	MYH14; EPHA1; MAPK13
Signaling to RAS	0.018	0.071	58.368	MAPK13
RHO GTPases activate ROCKs	0.018	0.071	58.368	MYH14
RHO GTPases activate CIT	0.018	0.071	58.368	MYH14
Sema4D induced cell migration and growth-cone collapse	0.019	0.071	55.293	MYH14
PD-1 signaling	0.021	0.071	52.526	PDCD1
RHO GTPases activate PAKs	0.021	0.071	52.526	MYH14
Nephrin family interactions	0.022	0.071	50.022	KIRREL2
Sema4D in semaphorin signaling	0.024	0.073	45.668	MYH14
Transcriptional regulation of pluripotent stem cells	0.029	0.087	36.208	EPHA1
Signaling to ERKs	0.032	0.087	32.809	MAPK13
NOD1/2 signaling pathway	0.032	0.087	32.809	MAPK13
G α (I) signaling events	0.038	0.098	7.050	LPAR5; GPSM3
EPH-ephrin mediated cell repulsion	0.047	0.113	21.855	EPHA1
Netrin-1 signaling	0.047	0.113	21.855	MAPK13

Table 8- This table shows the signaling pathways associated with the eQTLs from the cluster 1. An enrichment analysis was conducted using the Enrichr web platform, and results with a p-value <0.05 were considered significant. It also provides the eQTLs involved in each signaling pathway**.**

Table 9- Enrichment of EQTLs Cluster-2 associated with MetS-related SNP rs1803274 using Enrichr web application

Pathway	P-value	Adjusted P-value	Odds Ratio	Genes
Antigen processing: ubiquitination and proteasome degradation	0.0012	0,026	17.657	WSB1; ASB4; UBE2A
Class I MHC mediated antigen processing and presentation	0.0021	0.026	14.263	WSB1; ASB4; UBE2A
Neddylation	0.0115	0.066	14.008	WSB1; ASB4
Post-translational protein modification	0.0130	0.066	5.397	WSB1; SCG3; ASB4; UBE2A
Adaptive immune system	0.0132	0.066	7.194	WSB1; ASB4; UBE2A
WNT ligand biogenesis and trafficking	0.0180	0.074	61.418	VPS26A
Synthesis of active ubiquitin: roles of E1 And E2 enzymes	0.0208	0.074	52.936	UBE2A
RHOF GTPase cycle	0.0290	0.091	37.420	SOWAHC
Metabolism of proteins	0.0367	0.096	3.839	WSB1; SCG3; ASB4; UBE2A
E3 ubiquitin ligases ubiquitinated target	0.0385	0.096	27.876	UBE2A

proteins				
RHOB GTPase cycle	0.0472	0.107	22.532	SOWAHC
Protein ubiquitination	0.0519	0.108	20.422	UBE2A

Table 9- This table shows the signaling pathways associated with the eQTLs from the cluster 2. An enrichment analysis was conducted using the Enrichr web platform, and results with a p-value <0.05 were considered significant. It also provides the eQTLs involved in each signaling pathway**.**

Table 10- Enrichment of EQTLs Cluster-3 associated with MetS-related SNP rs1803274 using Enrichr web application.

**Pathway**	**P-valu e**	**Adjusted P-value**	**Odds Ratio**	**Genes**
Gene expression regulation in pancreatic β cells	0.010	0.054	116.78	GCK
Oxidative stress-induced gene expression via Nrf2	0.010	0.054	110.94	MAFK
Maturity onset diabetes of the young	0.012	0.054	92.43	GCK
Galactose metabolism	0.013	0.054	88.73	GCK
Pancreatic β-cell development regulation	0.016	0.054	71.53	GCK
Insulin biosynthesis and processing	0.020	0.054	55.42	PCSK2
Hexose transport	0.020	0.054	54.06	GCK

Type 1 diabetes mellitus	0.021	0.054	52.77	ICA1
Interactions of HIV Vpr protein with host cellular proteins	0.022	0.054	51.54	GCK
Amino sugar and nucleotide sugar metabolism	0.022	0.054	50.37	GCK
HNF3B pathway	0.022	0.054	50.37	GCK
DM2	0.025	0.054	45.22	GCK
JNK/MAPK pathway	0.026	0.054	42.60	GCK
Starch and sucrose metabolism	0.026	0.054	42.60	GCK

Table 10- This table shows the signaling pathways associated with the eQTLs from the cluster 3. An enrichment analysis was conducted using the Enrichr web platform, and results with a p-value <0.05 were considered significant. It also provides the eQTLs involved in each signaling pathway**.**

Table 11- Cluster 1 eQTLs associated with the MetS-related rs1803274-BuChe SNP

Gene name	Effect size	P-values
EPHA1	0.509	0.0017
TSPEAR	0.507	0.0018
EPCAM	0.484	0.0032
MYBPHL	0.463	0.0050
ELF4	0.456	0.0059
FAM83F	0.421	0.0119
PDCD1	0.416	0.0129
CD74	0.402	0.0168
KIRREL2	0.399	0.0173
MYH14	0.398	0.0177
C2CD4B	0.397	0.0182
SNORD19	0.393	0.0194
GALNT6	0.393	0.0196
LPAR5	0.388	0.0211
OR7E91P	0.385	0.0223
KLF13	0.385	0.0225
SMIM22	0.384	0.0226
GPSM3	0.376	0.0258
MAPK13	0.369	0.0292
SPINT1	0.352	0.0379

Table 11- This table shows the eQTLs of cluster 1 associated with the Smet-related rs1803274 BuChe SNP, displaying their effect sizes and p-values.

Table 12- Cluster 2 eQTLs associated with the MetS-related rs1803274-BuChe SNP

Gene name	Effect size	P-values
MIR375	0.444	0.0074
LRRC10B	0.442	0.0077
SCG3	0.401	0.0170
SOWAHC	-0.393	0.0195
VPS26A	-0.383	0.0230
MZF1	0.382	0.0235
FBXL6	0.377	0.0255
DENND11	0.367	0.0301
PROX1	0.360	0.0335
ASB4	0.352	0.0381
UBE2A	-0.350	0.0391
WSB1	0.345	0.0424
UBN2	0.339	0.0464

Table 12- This table shows the eQTLs of cluster 2 associated with the Smet-related rs1803274 BuChe SNP, displaying their effect sizes and p-values.

Table 13- Cluster 3 eQTLs associated with the MetS-related rs1803274-BuChe SNP

Gene name	Effect size	P values
PPM1N	0.468	0.0045
GCK	0.438	0.0085
PCSK2	0.383	0.0229
SLC66A3	-0.376	0.0260
MAFK	0.369	0.0290
REXO1	0.357	0.0352
TRAPPC9	0.354	0.0369
DUSP26	0.346	0.0417
TOR2A	0.339	0.0459
ICA1	0.337	0.0473

Table 13- Cluster 3 eQTLs associated with the MetS-related rs1803274-BuChe SNP. This table presents the genes (eQTLs) associated with the SNP rs1803274 specifically those computed in cluster 3 of the clustering analysis (see Figure 3), displaying their effect sizes and p-values.

Figures

Figure 1- Association between SNPs and MetS in PD patients (Additive model, Chi-square test).

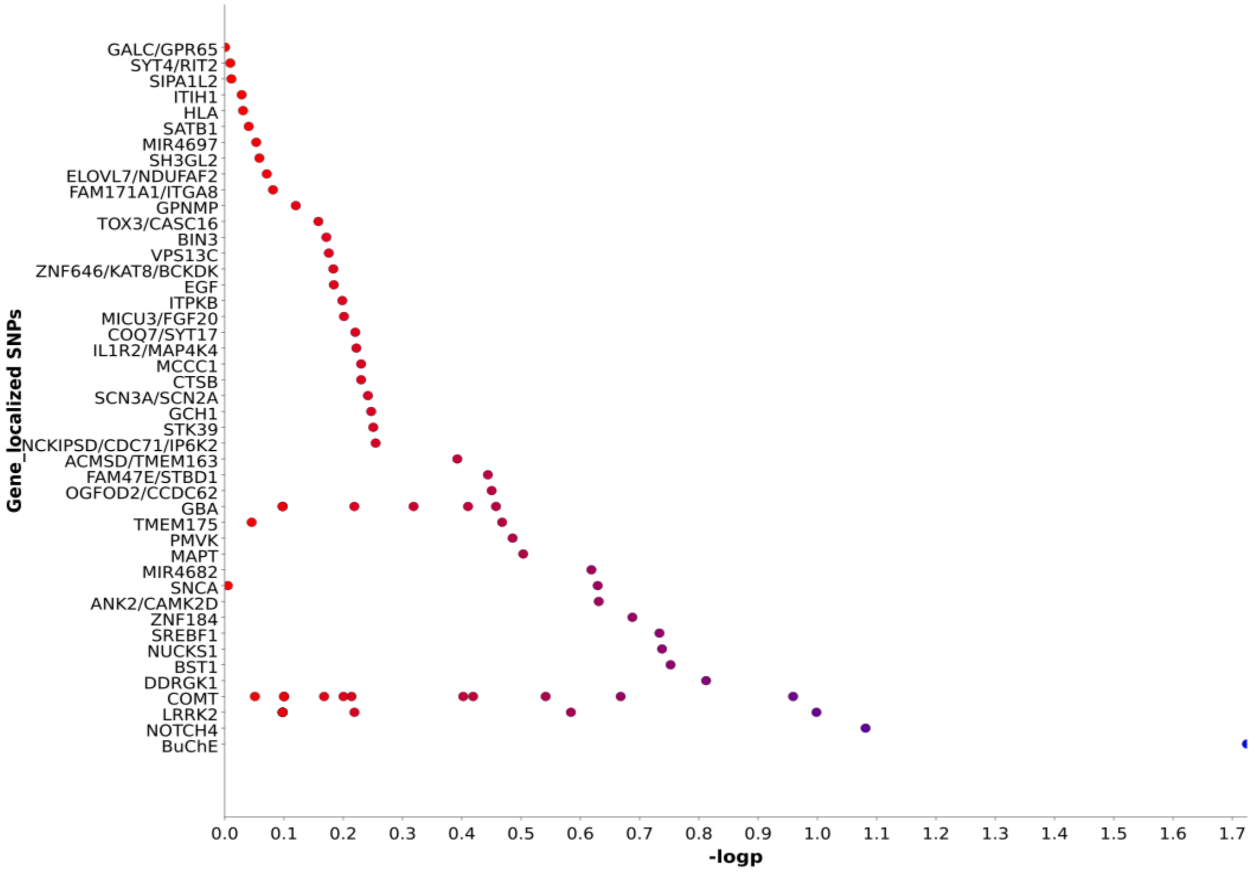

Figure 1- This figure shows the 72 SNPs associated with PD obtained from the PPMI database. Ordinates: genes-located SNPs. Abscissae: -log 10 (p). SNPs are represented by red dots (not associated with MetS, small -log 10 (p), p>0.05, N.S.) and blue dots (associated with MetS, large -log 10 (p), p< 0.05).

Figure 2 - Clustering analysis of the BuChe-located SNP associated eQTLs-based network.

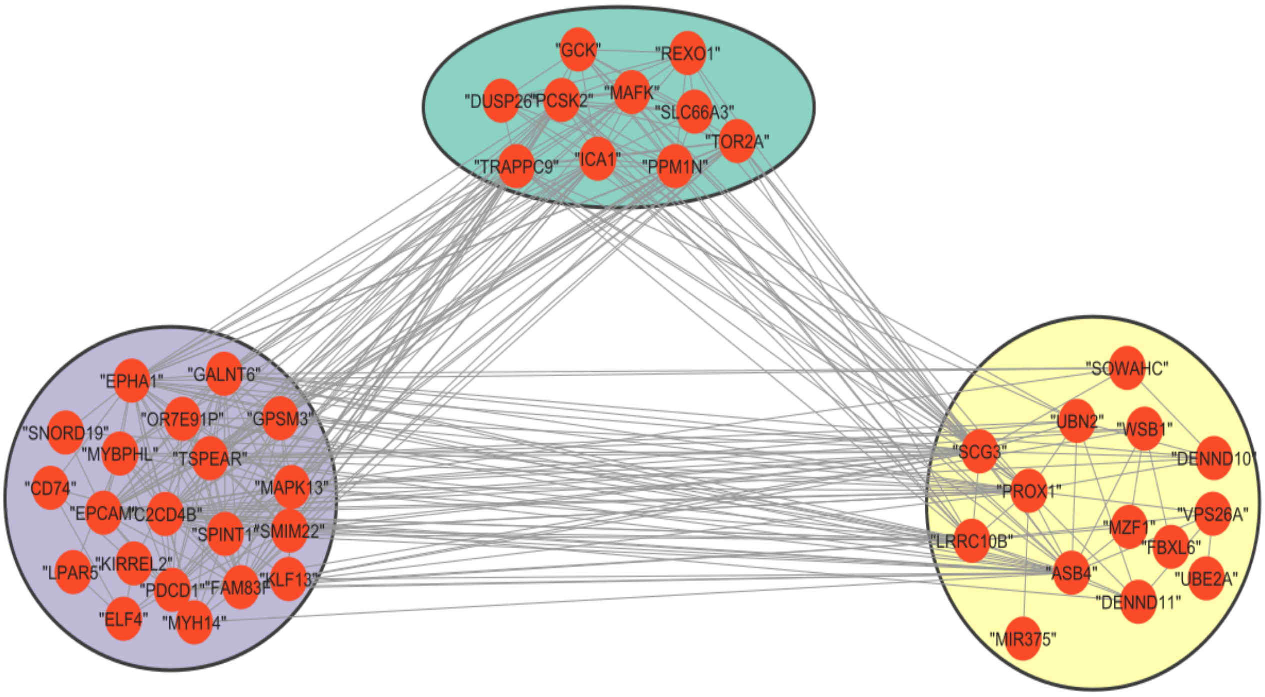

Figure 2- This figure shows the clusters formed using the clustering algorithm Cytoscape Autoannotate plugin. The eQTLs found associated with SNP rs1803274-BuChe and expressed distinctively according to SNP genotypes were divided into three clusters by the community cluster Glay annotation algorithm. The small circles within the large circles represent the nodes (eQTL, genes). Each large circle contains the elements of a cluster. Cluster 1 comprises 20 nodes and 98 edges, with a clustering coefficient of 0.75. It includes genes EPHA1, GALNT6, GPSM3, MAPK13, SMM22, KLF13, FAM83F, MYH14, ELF4, PDCD1, LPAR5, KIRREL2, SPINT1, C2CD4B, EPCAM, TSPEAR, MYBPHL, SNORD19, and OR7E91P. Cluster 2 comprises 14 elements with 29 edges and a clustering coefficient of 0.50. It includes genes SOWAHC, UBN2, WSB1, DENND10, SCG3, PROX1, MZF1, LRRC10B, ASB4, MIR375, DENND11, FBXL6, UBE2A, and VPS26A. Cluster 3, formed by 10 elements with 39 edges and a clustering coefficient of 0.88, includes genes REXO1, GCK, DUSP26, PCSK2, MAFK, SLC66A3, TOR2A, TRAPPC2, ICA1, and PPM1N.
